# Wie stark trifft die Corona-Pandemie die chirurgische Klinik eines universitären Maximalversorgers? Eine Analyse der ersten 7 Wochen

**DOI:** 10.1007/s00104-020-01255-y

**Published:** 2020-08-13

**Authors:** Nikolaus von Dercks, Daniel Seehofer, Matthias Steinert, Sebastian Krämer, Daniela Branzan, Arne Dietrich, Olaf Schürmann, Ines Gockel

**Affiliations:** 1 Stabsstelle Medizincontrolling, Universitätsklinikum Leipzig AöR, Liebigstraße 18, Haus B, 04103 Leipzig, Deutschland; 2 Klinik und Poliklinik für Viszeral‑, Transplantations‑, Thorax- und Gefäßchirurgie, Universitätsklinikum Leipzig AöR, Leipzig, Deutschland; 3 Department für Operative Medizin, Universitätsklinikum Leipzig AöR, Leipzig, Deutschland

**Keywords:** Corona-Pandemie, COVID-19-Krankenhausentlastungsgesetz, Erlös, Kompensation, Viszeralchirurgie, Corona pandemic, COVID-19 Hospital relief act, Revenue, Compensation, Visceral surgery

## Abstract

**Hintergrund:**

Die Corona-Pandemie stellt Krankenhäuser vor enorme finanzielle Herausforderungen. Am Beispiel einer Klinik für Viszeral‑, Transplantations‑, Thorax- und Gefäßchirurgie (VTTG) soll die Leistungsentwicklung in der stationären Versorgung der ersten 7 Wochen nach Beginn der gesetzgeberisch angeordneten Leistungsreduktion im Vorjahresvergleich sowie eine Bewertung der gesetzgeberisch festgelegten Kompensationsmaßnahmen bewertet werden.

**Methodik:**

Anhand der Leistungszahlen wird ein Vergleich des Zeitraumes vom 16.03. bis 03.05.2019 mit demselben Zeitraum 2020 durchgeführt. Veränderungen von Fallzahl, Case-Mix, Case-Mix-Index und Day-Mix-Index sowie der Belegungstage werden erfasst. Diesen Veränderungen werden die monetären Maßnahmen aus dem COVID-19-Krankenhausentlastungsgesetz gegenübergestellt und deren Auskömmlichkeit bewertet.

**Ergebnisse:**

Im Vergleich zum Vorjahr kommt es im Beobachtungszeitraum zu einem Rückgang der stationären Aufnahmen von 120 Patienten. Demzufolge waren ein Rückgang des Case-Mix um 370 Punkte und der Belegung um 1433 Tage zu verzeichnen. Über die gesamte VTTG ergibt sich ein Erlösrückgang von ca. 0,8 Mio. €, der durch die Leerbettenpauschale vollständig kompensiert wird. Die einzelnen Bereiche zeigen in Bezug auf die Kompensationsmechanismen ein heterogenes Bild mit einer Unterdeckung elektiver Bereiche bis zu 128.163 € in Bezug auf die stationären Leistungen für 7 Wochen.

**Diskussion:**

Die Maßnahmen des Gesetzgebers stellen eine wichtige Stütze zur wirtschaftlichen Absicherung deutscher Krankenhäuser dar. Die fehlende Differenzierung der Maßnahmen nach Fachrichtung führt für die VTTG zu einem heterogenen Bild in der Auskömmlichkeit und muss als Warnsignal insbesondere für elektive chirurgische Leistungserbringer gesehen werden.

## Abstract (en)

**Background:**

The corona pandemic poses enormous financial challenges for hospitals. Using the example of a clinic for visceral, transplantation, thorax and vascular surgery (VTTG), the performance development of inpatient care in the first 7 weeks in comparison to the previous year as well as an evaluation of the compensation measures laid down by law were evaluated.

**Methodology:**

Based on the performance figures, a comparison was made between the period from 16 March to 3 May 2019 and the same period in 2020. Changes in the number of cases, case mix, case mix index and day mix index as well as the bed occupancy days were recorded. The monetary measures from the COVID-19 Hospital Relief Act were applied to these changes and the adequacy was evaluated.

**Results:**

Compared to the previous year there was a decrease in inpatient admissions of 120 patients during the observation period. As a result there was a decrease of 370 points in case mix and 1433 days of occupancy. For the whole VTTG this resulted in a decrease in revenue of approximately 0.8 million €, which was completely compensated by the flat rate payment for empty beds. The individual areas of the VTTG showed a heterogeneous picture in relation to the compensation for the loss of revenue. Elective units in particular showed a shortfall up to 128,163 € with respect to inpatient treatment for 7 weeks.

**Conclusion:**

The measures taken by the Government are an important support for the economic security of German hospitals. The lack of differentiation of measures by specialty leads to a heterogeneous picture in compensation for VTTG revenues and has to be understood as an alarm signal especially for elective surgical healthcare.

## Hintergrund

Die Corona-Pandemie stellt das deutsche Gesundheitssystem vor extreme Herausforderungen [2, 6, 9]. Dabei sind die Leistungserbringer im ambulanten wie im stationären Sektor nachhaltig betroffen [3]. Vor diesem Hintergrund hat die Bundesregierung im März 2020 das „Gesetz zum Ausgleich COVID-19 bedingter finanzieller Belastungen der Krankenhäuser und weiterer Gesundheitseinrichtungen“ (COVID-19-Krankenhausentlastungsgesetz) beschlossen [5, 8]. Darin sind u. a. finanzielle Absicherungsmechanismen für medizinische Leistungserbringer enthalten. Für Krankenhäuser ist geregelt, dass sie für alle voll- und teilstationär aufgenommenen Patienten ab dem 01.04.2020 eine zusätzliche Vergütung von 50 € als Abgeltung für Preis- und Mengensteigerungen durch die Pandemie, insbesondere bei Schutzausrüstung, erhalten sollen [5]. Weiterhin wird der Pflegeentgeltwert vorübergehend von 146,55 € auf 185 € angehoben, der Aufbau zusätzlicher Intensivbetten wird gefördert und ein finanzieller Ausgleich für nicht belegte Betten wird gezahlt („Freihaltepauschale“, [4, 5, 8]). Gerade letzter Aspekt soll den Umstand berücksichtigen, dass planbare Operationen und andere Behandlungen verschoben werden müssen, um Infektionsrisiken zu vermeiden und Kapazitäten in den Krankenhäusern, insbesondere Intensivbetten, für infizierte Patienten zu schaffen [1, 11]. Kliniken mit einem hohen Anteil an elektiven Eingriffen sind demnach von den Maßnahmen besonders betroffen und auf die finanziellen Entlastungsmaßnahmen angewiesen. Im Rahmen einer Verordnung justierte das Bundesgesundheitsministerium die zunächst für alle Krankenhäuser pauschal angesetzte Freihaltepauschale von 560 € in eine differenzierte Ausgleichszahlung von 360–760 € nach [12]. Mit der Differenzierung soll den unterschiedlichen Kostenstrukturen der Krankenhäuser besser Rechnung getragen werden.

Im Rahmen des Zweiten Gesetzes zum Schutz der Bevölkerung bei einer epidemischen Lage von nationaler Tragweite sind weitere Mechanismen zur finanziellen Absicherung medizinischer Leistungserbringer geregelt [15]. Unter anderem ist ein Zusatzentgelt für die Testung auf eine SARS-CoV2-Infektion vorgesehen und Abrechnungsprüfungen durch die Kostenträger sind auch für 2021 nur begrenzt möglich.

Ziel dieser Arbeit ist es, am Beispiel der Klinik und Poliklinik für Viszeral‑, Transplantations‑, Thorax- und Gefäßchirurgie eines universitären Maximalversorgers nach den ersten 7 Wochen der restriktiven Maßnahmen im Rahmen der Corona-Pandemie die Leistungszahlen für stationäre Behandlungen im Vorjahresvergleich zu betrachten. Darüber hinaus soll eine Abschätzung zur Auskömmlichkeit der finanziellen Ausgleiche getroffen werden.

## Studiendesign und Untersuchungsmethoden

An der Klinik für Viszeral‑, Transplantations‑, Thorax- und Gefäßchirurgie (VTTG) werden jedes Jahr über 3000 Patienten stationär versorgt. Diese verteilen sich auf fünf Bereiche: Viszeralchirurgie (VisCh), hepatobiliäre und viszerale Transplantationschirurgie (VTxCh), Gefäßchriurgie (GefCh), Thoraxchirurgie (ThoCh) und bariatrische Chirurgie (BarCh). Es soll der Zeitraum der pandemiebedingten Restriktionen ([6]; 16.03.2020 bis 03.05.2020) mit exakt dem Vorjahreszeitraum (16.03.2019 bis 03.05.2019) verglichen werden. Dabei ist das Aufnahmedatum der Referenzzeitpunkt. Im Rahmen einer deskriptiven Analyse erfolgt die Aufarbeitung der Leistungszahlen der Klinik sowie eine Bewertung der Belegtage anhand der gesetzlich vorgesehenen Kompensationszahlungen 2020. Es wird neben den gängigen Größen Fallzahl, Case-Mix (CM, Summe der Bewertungsrelationen aller Fälle) und Case-Mix-Index (CMI, Summe der Bewertungsrelationen dividiert durch die Fallzahl [durchschnittliche Bewertungsrelation pro Fall]) auch der sog. Day-Mix-Index (DMI) berücksichtigt, also der CM eines Behandlungsfalls dividiert durch dessen Belegungstage. Weiterhin werden die Erlöse für die stationären Behandlungsfälle dargestellt und verglichen, da aufgrund der Ausgliederung der Pflegekosten ab 2020 ein direkter Vergleich von Relativgewichten alleine nicht zielführend wäre. Patienten im Jahr 2020, die über den Betrachtungszeitrum hinaus noch stationär behandelt werden, werden mit ihrer aktuellen DRG („diagnosis related groups“) bewertet. Begleitpersonen werden bei der Berechnung der Leistungszahlen nicht berücksichtigt. Privatpatienten werden nach DRGs abgerechnet und sind mit berücksichtigt. Zur Berechnung des Pflegeentgelts erfolgt die individuelle Multiplikation von Belegungstagen, Pflegerelativgewicht und Pflegeentgeltwert. Letzter beträgt bis zum 31.03.2020 146,55 € und ab 01.04.2020 185,00 €. Dies wurde bei den Monatsüberliegern von März nach April 2020 entsprechend anteilig berücksichtigt. Als Ausgleich für den Leerstand von Krankenhausbetten sind für die VTTG 660 € anzusetzen.

Der Landesbasisfallwert (LBFW) betrug für Sachsen 2019 3528,65 € und 2020 3663,09 €. Zum Vergleich des DMI-Äquivalents, das aus der Kompensation ausbleibender Belegungstage resultiert, wird der DMI vor dem Beobachtungszeitraum 2020 herangezogen, wobei Jahresüberlieger von 2019 nach 2020 nicht betrachtet werden.

## Ergebnisse

**Figure 1:**
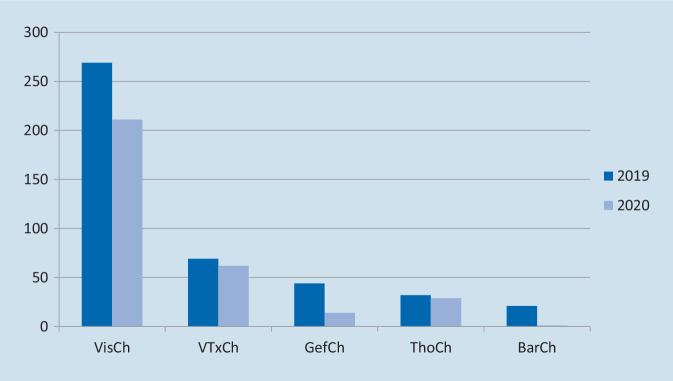

Im Untersuchungszeitraum 2019 wurden 435 Personen an der VTTG aufgenommen ([434 Patienten, 1 Begleitperson] 269 VisCh, 69 VTxCh, 44 GefCh, 32 ThoCh, 21 BarCh). Im gleichen Zeitraum 2020 wurden 317 Patienten aufgenommen ([314 Patienten, 3 Begleitpersonen] 211 VisCh, 62 VTxCh, 14 GefCh, 29 ThoCh, 1 BarCh). Die Abb. 1 zeigt die Relationen beider Zeiträume. Das bedeutet einen Rückgang der Fallzahl von insgesamt 27 % (VisCh: 22 %, VTxCh: 10 %, GefCh: 68 %, ThoCh: 9 %, BarCh: 95 %).

**Figure 2:**
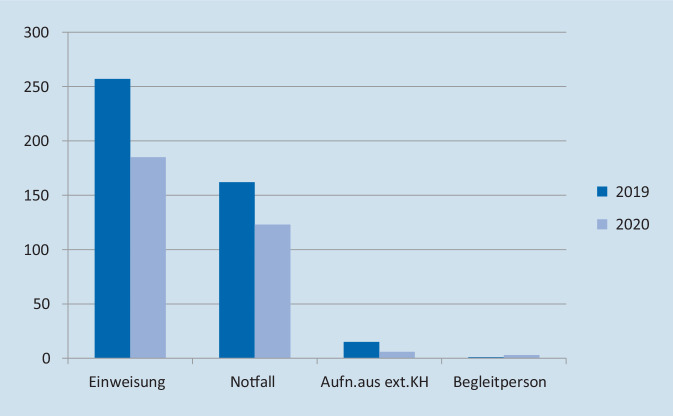

Die Differenzierung nach der Aufnahmeart ist in Abb. 2 und Tab. 1 dargestellt. Demnach lässt sich ein Rückgang der elektiven Einweisungen um 72 Fälle (28 %) feststellen. Die Verlegungen aus externen Krankenhäusern gingen um 60 % zurück, Notfälle um 24 %. Der Anstieg der Begleitpersonen von 1 (2019) auf 3 im Jahr 2020 ist besonderen Umständen beim Betreuungsaufwand einzelner Patienten geschuldet und soll nicht weiter analysiert werden.

**Table 1:** 

Bereiche	Aufnahmejahr		∆ (%)
	2019	2020	
*VisCh*	*269*	*211*	*−22*
Aufn. aus ext. KH	11	5	−55
Begleitperson	1	3	200
Einweisung	125	101	−19
Notfall	132	102	−23
*VTxCh*	*69*	*62*	*−10*
Aufn.aus ext. KH	3	–	−100
Einweisung	57	52	−9
Notfall	9	10	11
*GefCh*	*44*	*14*	*−68*
Aufn. aus ext. KH	1	–	−100
Einweisung	23	5	−78
Notfall	20	9	−55
*ThoCh*	*32*	*29*	*−9*
Aufn. aus ext. KH	–	1	100
Einweisung	31	26	−16
Notfall	1	2	100
*BarCh*	*21*	*1*	*−95*
Einweisung	21	1	−95
**Aufnahmen gesamt**	**435**	**317**	**−27**

*Aufn. aus ext. KH* Aufnahme aus externen Krankenhaus, *VisCh* Viszeralchirurgie, *VTxCh* hepatobiliäre und Transplantationschirurgie, *GefCh* Gefäßchriurgie, *ThoCh* Thoraxchirurgie, *BarCh* bariatrische Chirurgie

Die 434 Behandlungsfälle 2019 (ohne Begleitpersonen) generierten 3709 Behandlungstage bei einem CM von 968 Punkten, einem CMI von 2,231 und einem DMI von 0,261. Daraus resultiert auf Basis des Landesbasisfallwerts von 2019 ein Erlös von 3.416.248 € (Tab. 2). Demgegenüber resultieren bei den im Zeitraum 2020 behandelten Patienten 2276 Behandlungstage und ein CM von 598 Punkten mit zusätzlich 2386 Pflegerelativgewichten. Es ergibt sich für 2020 ein CMI von 1,905 und ein DMI (ohne Pflege) von 0,263. Der DRG-Erlös inkl. Pflegeerlöse für den Beobachtungszeitraum 2020 beträgt 2.616.344 €, was einem Rückgang gegenüber dem Vorjahr von 799.905 € bzw. 23 % entspricht. Dabei ist der erhöhte Pflegeentgeltwert ab 01.04.2020 für den Beobachtungszeitraum 2020 bereits berücksichtigt und beträgt anteilig 75.249 €.

**Table 2:** 

Bereich	Erlöse 2019 (€)	Erlöse 2020 (€)	∆ (€)
VisCh	1.746.477,09	1.534.477,60	211.999
VTxCh	794.454,38	678.501,83	115.953
GefCh	470.026,77	122.043,68	347.983
ThoCh	254.602,68	274.171,61	19.569
BarCh	150.687,47	7149,12	143.538
*Gesamtergebnis*	*3.416.248,38*	*2.616.343,84*	*799.905*

*VisCh* Viszeralchirurgie, *VTxCh* hepatobiliäre und Transplantationschirurgie, *GefCh* Gefäßchriurgie, *ThoCh* Thoraxchirurgie, *BarCh* bariatrische Chirurgie

**Figure 3:**
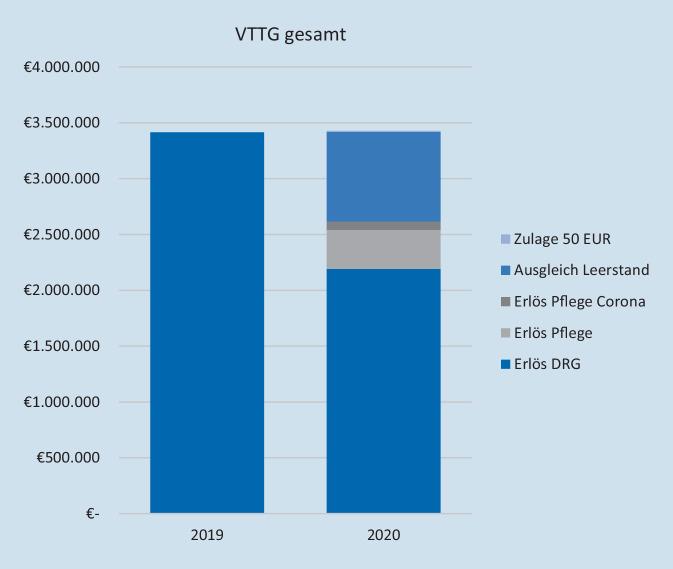

Nicht erbrachte Belegungstage der VTTG im jahr 2020 gegenüber dem Vorjahr werden mit 660 € pro Tag kompensiert. Im Beobachtungszeitraum 2020 waren 1433 Belegungstage weniger als im Vorjahreszeitraum zu verzeichnen. Daraus ergibt sich eine Ausgleichsvergütung von 945.780 €. Aus diesen Betrachtungen resultiert unter Berücksichtigung monetär kompensierter Leerstände, der 50-€-Zulage pro stationärem Fall sowie des erhöhten Pflegeentgeltwertes eine vollständige Kompensation des Erlösrückganges gegenüber dem Vorjahr (Abb. 3).

**Figure 4:**
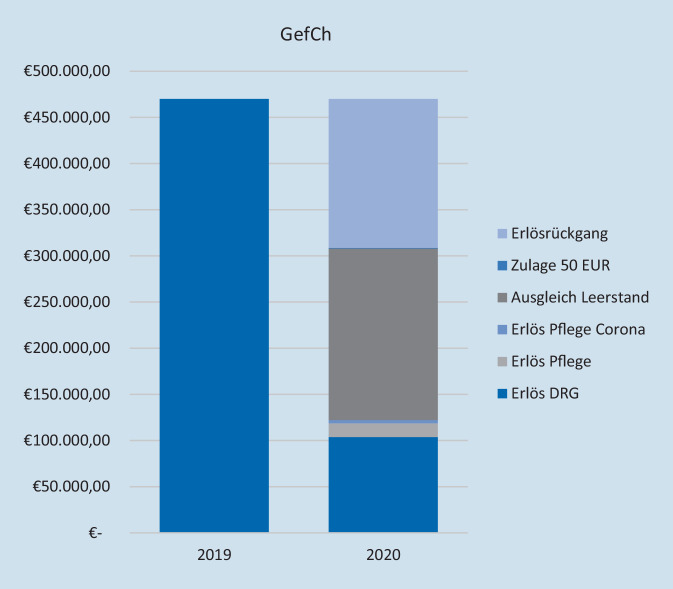

**Figure 5:**
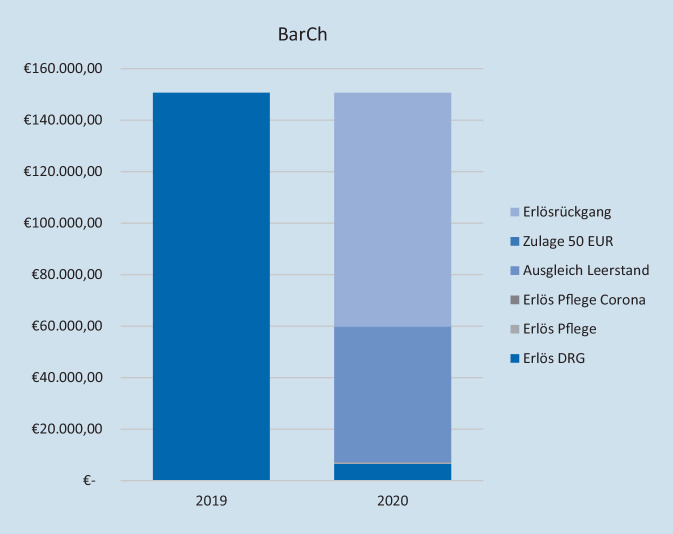

Auf der Ebene der Bereiche der Klinik VTTG ist die Kompensation heterogen. Während aufgrund des veränderten Leistungsgeschehens in VisCh, VTxCh und ThoCh verbunden mit finanzieller Entschädigung für ausgefallene Leistungen eine Überkompensation erzielt wird (VisCh: 17 %, VTxCh: 8 %, ThoCh: 8 %), reichen die finanziellen Unterstützungsmaßnahmen für GefCh und BarCh nicht aus. Es resultiert für die beiden Bereiche trotz Kompensationsmaßnahmen ein Erlösrückgang von 27 % (GefCh, Abb. 4) und 54 % (BarCh, Abb. 5). Im Falle der Gefäßchirurgie ist neben den Absagen elektiver Patienten der Umstand eines (gleichzeitigen) 14-tägigen Ausfalls von 3 Operateuren (75 %) durch Quarantänemaßnahmen zu nennen. Diese Einschränkung betraf die anderen Bereiche der VTTG nicht.

Die auskömmliche Kompensation der Bereiche VisCh, VTxCh und ThoCh resultiert nicht zuletzt aus der Zunahme der Fallschwere der behandelten Patienten während des Beobachtungszeitraumes 2020. Der Vergleich zwischen dem CMI im Zeitraum vor den Corona-Restriktionen 2020 und währenddessen (Beobachtungszeitraum) ist in Tab. 3 dargestellt.

**Table 3:** 

Bereich VTTG	CMI 2020 vor Corona	CMI Corona	∆ (%)
VisCh	1,237	1,653	34
VTxCh	2,265	2,550	13
GefCh	2,393	2,023	−15
ThoCh	1,882	2,281	21
BarCh	1,749	1,779	2
*Gesamtergebnis*	*1,621*	*1,905*	*18*

*CMI *Case-Mix-Index, *VTTG* Klinik für Viszeral‑, Transplantations‑, Thorax- und Gefäßchirurgie, *VisCh* Viszeralchirurgie, *VTxCh* hepatobiliäre und Transplantationschirurgie, *GefCh* Gefäßchriurgie, *ThoCh* Thoraxchirurgie, *BarCh* bariatrische Chirurgie

Der höhere CMI während der pandemiebedingten Restriktionen bedeutet bei 314 Behandlungsfällen einen Mehrerlös von 327.344 € gegenüber dem CMI der Monate davor. Analog lässt sich auch ein Anstieg des DMI aufgrund des veränderten Leistungsspektrums während der Restriktionen nachweisen (DMI vor Corona-Restriktionen: 0,215; DMI während Restriktionen: 0,263).

## Diskussion

Die Corona-Pandemie bedeutet für das deutsche wie das weltweite Gesundheitswesen eine enorme medizinische und wirtschaftliche Herausforderung [2, 6, 9]. Die Bundes- und Landespolitik unterstützt mit einem großen Maßnahmenkatalog, kann aber allenfalls auf geringe Empirie zurückgreifen [5]. Zudem kommen der zeitliche und der öffentliche Druck, mit dem Entscheidungen von der Politik erwartet wurden. In diesem Zuge trat am 28.03. das COVID-19-Krankenhausentlastungsgesetz in Kraft [8]. Darin werden zahlreiche monetäre Kompensationsmechanismen für Krankenhäuser geregelt, die meist rückwirkend Geltung haben. Diese Arbeit vergleicht den Zeitraum seit Beginn der restriktiven Maßnahmen an der Klinik (Aussetzen elektiver Operationen, Reduktion stationärer Kapazitäten u. a.) mit dem gleichen Zeitraum des Vorjahres. Dieser bot keine Auffälligkeiten in den klinischen Leistungszahlen, sodass er als Referenz als geeignet angesehen werden kann. Die Wahl des Zeitraumes 2020 beruht auf der Maßgabe eines Beschlusses, der von der Bundeskanzlerin mit den Ministerpräsidenten der Länder am 12.03.2020 getroffen wurde [6]. Darin heißt es, dass „grundsätzlich alle planbaren Aufnahmen, Operationen und Eingriffe in allen Krankenhäusern ab Montag [16.03.2020] auf unbestimmte Zeit verschoben und ausgesetzt werden“ [6].

Die Deutsche Gesellschaft für Chirurgie (DGHC) unterstützt in ihrer Stellungnahme vom 24.03.2020 die Bemühungen zur Einschränkung der Pandemie [10]. Entsprechend der Bund-Länder-Entscheidung reduzierte auch die VTTG ihr Operationsprogramm ab Montag, dem 16.03.2020.

Im Anschluss an diese Entscheidung war sowohl ein Rückgang der Einweisungen und Zuweisungen aus anderen Krankenhäusern als auch der Notfallpatienten zu verzeichnen. Gerade letzter Umstand lässt sich auch in der Kardiologie und anderen klinischen Bereichen beobachten und ist auf die Besorgnis der Patienten zurückzuführen, sich im Krankenhaus womöglich mit SARS-CoV‑2 zu infizieren [7]. Bereits in ihrer 2. Ad-hoc-Stellungnahme hat die Leopoldina auf die Notwendigkeit hingewiesen, dass relevante Diagnosen auch in Zeiten der Pandemie weiterhin frühzeitig gestellt werden müssen [14]. Erwartungsgemäß ist der Patientenrückgang in dem rein elektiven Bereich der Bariatrie mit 95 % am deutlichsten.

Regulär weiter operiert wurden vor allem Tumorpatienten und Notfälle, was sich im Anstieg des CMI und DMI widerspiegelt. Gleichzeitig wurde das elektive Operationsprogramm nahezu vollständig eingestellt. Somit bestand die Möglichkeit, die Operationssäle der elektiven Bereiche mit zu nutzen, sodass insbesondere Tumorpatienten zeitnäher versorgt werden konnten. Damit wurde auch dem Aufruf der 4. Ad-hoc-Stellungnahme der Leopoldina gerecht, onkologische und chronisch kranke Patienten nicht aus dem Fokus zu verlieren [12]. Die relative Zunahme an onkologischen und Transplantationsfällen und die damit verbundene Steigerung der Fallschwere (CMI) stellt dabei für sich alleine bereits einen internen finanziellen Kompensationsmechanismus dar. Personalkapazitäten für die Operationen und Behandlung dieser Patienten konnten während des Beobachtungszeitraumes konstant sichergestellt werden.

Insgesamt ist durch die Restriktionen ein Fallzahlrückgang von 27 % bzw. ein Erlösrückgang von 23 % im Beobachtungszeitraum zu verzeichnen. Dazu führten neben den politischen Verordnungen auch Quarantänemaßnahmen einzelner ärztlicher Mitarbeiter – insbesondere der GefCh –, sodass sich der Leistungsrückgang auch dadurch erklären lässt.

Durch die aktuelle Gesetzgebung wird hier gegengesteuert. Das Gros des Erlösrückganges sollte durch eine Pauschale von 560 € für jedes Bett, das im Zeitraum vom 16.03. bis zum 30.09.2020 nicht belegt wird, ausgeglichen werden [8]. Der Ausgleich soll aus der Liquiditätsreserve des Gesundheitsfonds bezahlt werden [5, 8]. Dabei erfolgte zunächst keine Differenzierung nach Krankenhausgröße, Hauptabteilung oder einem anderen Schlüssel. Schröer et al. empfahlen daher die Orientierung der Pauschale am CMI, um dem Schweregrad bzw. der dafür vorgehaltenen Leistung gerecht zu werden [10]. Durch Verordnung des Bundesgesundheitsministeriums wurde im Verlauf nachgebessert und eine Pauschale auf Basis des DMI festgelegt [13].

Weiterhin stellt die Aufwertung des Pflegeentgeltwertes auf 185 € für den Beobachtungszeitraum einen, wenn auch nur geringen Kompensationsmechanismus dar. Grundlegend sind die Pflegeerlöse als Abschlagszahlung auf das Pflegebudget zu verstehen. Das bedeutet, dass alle Pflegeerlöse zwar akut liquiditätswirksam sind, jedoch letztendlich mit dem testierten und verhandelten Pflegebudget abgeglichen werden.

Weitere Kompensationsmaßnahmen des COVID-19-Krankenhausentlastungsgesetzes wie z. B. das Aussetzen des Fixkostendegressionsabschlags für 2020 sind hilfreich [8], auf einen einzelnen Bereich und kurzen Zeitraum aber nicht ohne Weiteres geldwert umzurechnen. Die in § 21 Abs. 6 KHG geregelte Corona-Mehrkostenpauschale von 50 € pro Patient fällt bei der vorliegenden Betrachtung kaum ins Gewicht [10]. Anhand einer aktuellen Untersuchung des DKI zeigte sich aber, dass für 90 % der Krankenhäuser der Preis- sowie Mengenanstieg für Schutzmaßnahmen nicht auskömmlich durch 50 € pro Patient kompensiert werden [2]. Außerdem sind Erlösausfälle in den Hochschulambulanzen überhaupt nicht adressiert, ein Umstand, der sich bei der bereits vor der Pandemie bestehenden Unterdeckung nun weiter verschärft [3]. Erfreulich ist allerdings die Verpflichtung der Kostenträger durch den Gesetzgeber zu einer verkürzten Zahlungsfrist von 5 Tagen nach Zugang der Krankenhausrechnung. Jedoch unterstützt auch dieser Schritt lediglich die Liquidität der Krankenhäuser und stellt kein zusätzliches Geld zur Verfügung [5]. Auch die verringerte Prüfquote für den Medizinischen Dienst (MD) und das Aussetzen der Sanktionszahlungen für beanstandete Fälle durch den MD ist vorteilhaft [5].

Zusammenfassend ist festzuhalten, dass es dem Gesetzgeber gelungen ist, sichtbare wirtschaftliche Unterstützung für die Krankenhäuser zu gewähren [9]. Die aufgeführten Mechanismen sichern Erlösrückgänge viszeralchirurgischer Kliniken aber nicht vollständig ab. Das liegt aus Sicht der Autoren maßgeblich an der noch immer nicht adäquaten Ausgestaltung der Kompensationszahlung für Leerbetten [9], die keine Differenzierung nach elektiven, semielektiven und Notfallbehandlungen aufweist. Auch finden quarantänebedingte Personalausfälle keine Berücksichtigung. Weiterhin ist die fast vollständige Einstellung des elektiven Ambulanzgeschehens und daraus resultierend auch die Reduktion zumindest kurz- und mittelfristiger Einweisungen äußerst bedrohlich [3, 9].

Hoffnung gibt das Zweite Gesetz zum Schutz der Bevölkerung bei einer epidemischen Lage von nationaler Tragweite [15]. Darin wird u. a. das Prüfgeschehen durch die Kostenträger bis Ende 2021 weiterhin restriktiv geregelt und ein Zusatzentgelt für die Testung auf das Coronavirus geregelt.

## Limitationen

Eine Bewertung zur Auskömmlichkeit der verschiedenen Kompensationsmechanismen ist nach dem relativ kurzen Zeitraum und der heterogenen Verteilung auf die untersuchten Bereiche abschließend nicht möglich. Außerdem werden in der vorliegenden Arbeit nur stationäre Fälle betrachtet. Die Auswirkung der pandemiebedingten Restriktionen auf die (Hochschul‑)Ambulanzen und damit ausbleibende Erlöse und Einweisungen lassen sich nicht suffizient abschätzen.

Die Aussage für ein gesamtes Klinikum ist aus der Betrachtung einzelner Bereiche nicht möglich.

## Schlussfolgerung bzw. „Fazit für die Praxis“

- Die Bundesregierung reagiert mit einem Maßnahmenpaket zur wirtschaftlichen Unterstützung der Krankenhäuser auf die Corona-Pandemie.
- Die Kompensationsmechanismen haben unterschiedliche Ansatzpunkte.
- Elektive und nichtelektive Bereiche der Klinik für Viszeral‑, Transplantations‑, Thorax- und Gefäßchirurgie (VTTG) sind aufgrund der vorgeschriebenen Verschiebung elektiver Behandlungen unterschiedlich stark von der Pandemie betroffen.
- Die wirtschaftliche Unterstützung durch die jüngste Gesetzgebung berücksichtigt nur die stationäre Patientenversorgung.
